# New fossils imply a deeper origin of modern birds in the Mesozoic

**DOI:** 10.1093/nsr/nwaf238

**Published:** 2025-06-09

**Authors:** Shaoyuan Wu, Ziqi Tao, Liang Liu, Charles R Marshall, Scott V Edwards, Zhonghe Zhou, Frank E Rheindt

**Affiliations:** Jiangsu Key Laboratory of Phylogenomics & Comparative Genomics, School of Life Sciences, Jiangsu Normal University, Xuzhou 221116, China; Linguistic Science Laboratory of Jiangsu Normal University, Laboratory of Philosophy and Social Sciences at Universities in Jiangsu Province, Xuzhou 221009, China; School of Linguistic Sciences and Arts, Jiangsu Normal University, Xuzhou 221009, China; Jiangsu Key Laboratory of Phylogenomics & Comparative Genomics, School of Life Sciences, Jiangsu Normal University, Xuzhou 221116, China; Xuzhou Central Hospital, Xuzhou 221003, China; Department of Statistics & Institute of Bioinformatics, University of Georgia, Athens, GA 30606, USA; Department of Integrative Biology, University of California Museum of Paleontology, University of California, Berkeley, CA 94720, USA; Department of Organismic and Evolutionary Biology, Museum of Comparative Zoology, Harvard University, Cambridge, MA 02138, USA; Key Laboratory of Vertebrate Evolution and Human Origins, Institute of Vertebrate Paleontology and Paleoanthropology, Chinese Academy of Sciences, Beijing 100044, China; Department of Biological Sciences, National University of Singapore, Singapore 117543, Singapore

**Keywords:** Mesozoic, K/Pg boundary, bird fossils, modern bird diversification, molecular clock

## Abstract

Macroevolutionary forces, such as rare catastrophes, have repeatedly disrupted and reset the evolutionary trajectories of Earth's major organismal groups. The Cretaceous–Paleogene (K/Pg) extinction event, approximately 66 Ma, resulted in the demise of ∼75% of all species at the time, yet despite its magnitude, many major organismal lineages successfully passed through this mass extinction. The evolutionary origins of modern birds (crown-group Aves) remain a subject of substantial debate, as they are often thought to have undergone their primary diversification following the K/Pg boundary. In this review, we summarize the various approaches that have been applied to understanding the timing of avian diversification. We examine the inferred divergence times derived from modern phylogenomic studies based on datasets comprising 50 to over 300 whole genomes. Additionally, we evaluate the factors contributing to the continued discrepancies in divergence time estimates. Furthermore, we discuss significant new fossil discoveries from the Late Jurassic and Late Cretaceous periods that reshape our understanding of key evolutionary events in early avian diversification. Taken together, the paleontological evidence increasingly supports a Cretaceous origin for many extant bird lineages, with the major burst of ordinal diversification likely occurring prior to the K/Pg boundary—concurrent with the early radiations of flowering plants, pollinating insects, mammals, fishes and other groups that characterized the Cretaceous Angiosperm Terrestrial Revolution.

## CONTROVERSY HAS SURROUNDED THE TIMING OF AVIAN DIVERSIFICATION

**Figure 1.**
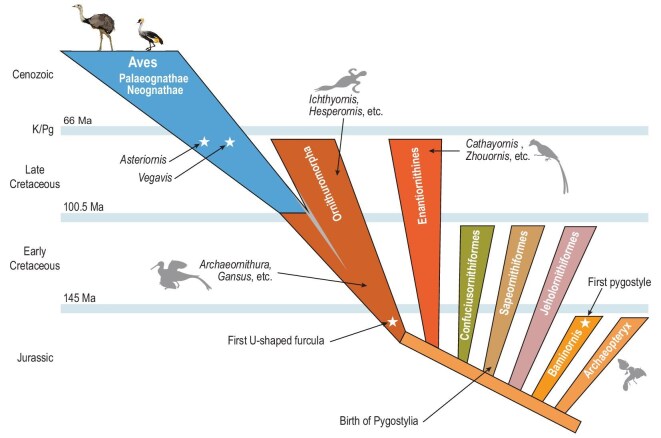
New view of early bird diversification, showing the emergence of pygostyle and U-shaped furcula in the Late Jurassic and the occurrence of crown-group birds (Aves) in the Cretaceous. The phylogenetic relationships of birds depicted in the figure are based on Wang *et al.* [20]. The age of crown-group birds is based on Brocklehurst and Field [15], while the gray shading represents the estimated age of modern birds according to Wu *et al.* [14]. The bird silhouettes in the figure are adapted from Wang *et al.* [20] and Wu *et al.* [14].

Few major organismal groups have seen as much controversy surrounding the timing of their origin as birds [1–16]. The precursors of modern birds (the stem-group Aves) date back to *Archaeopteryx* at ∼150 Ma [17,18]. Entire radiations of stem-group birds, such as the Ornithuromorpha and Enantiornithes (Fig. 1), have been characterized from the Cretaceous fossil record [19–21]. However, the origination of the most recent ancestors of modern living birds (crown-group Aves) has been intensely debated since the 1990s, with two major schools of thought.

Most studies from the early days of the genetic era, prior to the next generation sequencing (NGS) revolution, including research on proteins, DNA–DNA hybridization and single gene studies, pointed to a Cretaceous diversification of modern bird orders long before the catastrophic event at the Cretaceous–Paleogene (K/Pg) boundary (∼66 Ma) that led to the demise of non-avian dinosaurs and many other groups [3,4,6]. This hypothesis suggested that crown-group birds passed through the K/Pg extinction relatively unscathed. Further support for this view came from extensive biogeographic datasets focusing on principal events of continental break-up prior to the K/Pg boundary, especially the division of southern hemisphere Gondwanaland into multiple continents [1,5]. These studies revealed that pre-K/Pg continental shifts are largely consistent with the idiosyncratic biogeographic distribution of modern bird orders across different parts of the world.

The initial majority view of a Late Cretaceous avian diversification was gradually eroded by paleontological arguments that highlighted the near-complete absence of crown-group bird fossils prior to the K/Pg boundary, which stands in contrast to a steadily rising number of Late Cretaceous avian fossils outside crown-group Aves (e.g. Enantiornithes, Ornithuromorpha) [2,7]. Before the new millennium, claims of Cretaceous avian crown-group fossils largely consisted of fragmentary bones of controversial affinity [7,8]. This lack of unequivocal crown-group birds in the Cretaceous led to Feduccia's ‘explosive post-K/Pg radiation’ model of modern birds [2,7]. However, more recently, two avian crown-group fossils of firm taxonomic attribution were discovered from the latest Cretaceous deposits, suggesting a pre-K/Pg time of origin of at least some major clades of living birds. These include *Vegavis iaai*, an anseriform (i.e. duck and goose relative) from Antarctica at ∼68 Ma [9], but whose assignment to crown-group Aves has been called into question [22–24], and a second fossil, *Asteriornis maastrichtensis*, an unequivocal basal member of crown Aves (e.g. as part of Galloanseres or Paleognathae) from ∼67 Ma in Belgium [10,25,26]. Here we discuss the most recent fossil discoveries and progress in phylogenomic studies that add strength to the idea of a Cretaceous origin of modern bird clades (Fig. 1).

## EARLY GENOMIC ANALYSES FAVOR THE NEOAVIAN EXPLOSION MODEL

The NGS revolution has seen the application of genome-wide DNA datasets to the question of the timing of avian diversification. Two early landmark studies with whole genomes for roughly 50 and 200 avian genera from most modern bird orders, respectively, concluded that there was an explosive radiation of the Neoaves, which make up ∼95% of all modern birds, beginning at about the time of the K/Pg boundary [11,12]. These studies offered some reconciliation of prior disagreements by also concluding that the initial crown-group diversification occurred before the K/Pg boundary [11,12]. This new view was further supported by more recent work expanding the number of bird genomes to over 360 species [16]. The concurrence of these studies, each based on dozens to hundreds of genomes, instills confidence that most modern bird orders, at least those belonging to Neoaves, originated during an evolutionary frenzy following the K/Pg catastrophe. Their results are consistent with the idea that many of the ecological niches suddenly left vacant by the K/Pg event were subsequently re-occupied by the diversifying Neoaves, an intuitively compelling explanation for Earth's modern avian diversity. At the same time, these studies promote a scenario putting the avian radiation at odds with many other radiations that commenced long before the K/Pg boundary, such as mammals, flowering plants, fishes and pollinating insects etc. [14,27–34]. However, a recent phylogenomic study casts doubt on the Neoavian post-K/Pg explosion hypothesis [14]. The new analysis of over 120 whole genomes and multiple fossil constraints did not find support for a post-K/Pg Neoavian explosion [14], instead placing the emergence of ∼24 Neoavian orders within the Late Cretaceous, largely between ∼66 and 85 Ma. Thus, despite the growing quantity of NGS data, we still have no resolution on when the major radiation of birds occurred.

## INCOMPLETE FOSSIL RECORD—THE ACHILLES HEEL OF TEMPORAL CALIBRATION

There are multiple methodological issues associated with modeling and analyzing the genomic data that influence inferred divergence times [35], but fossil calibration is the Achilles heel of timetree estimation, especially in birds. The greatest challenge lies in the incompleteness of the fossil record, such that the oldest fossil of a group will generally be younger than the group's actual time of origin, thereby providing only a minimum estimate. Hence, the values selected as a bound on maximum bounds of various key divergence events have a major impact on our estimates of when a lineage such as crown-group birds began to diversify, and when it underwent its most extensive radiation.

Frustratingly, there are currently no rigorous or failsafe methods to address the bias generated from the incomplete fossil record [36]. However, two basic approaches have been widely embraced to deal with the issue. The first involves assigning maximum constraints for the age of a node, either by using taphonomic control groups or by relying on the oldest known fossils from the sister lineages of the group of interest. A taphonomic control group is a group that is expected to be co-preserved with the lineage of interest. Thus, when fossils of the control group are found without fossils of the group of interest, it is inferred that the group of interest had not yet evolved. However, there is no standard way for selecting appropriate control groups, and no one has yet quantified the likelihood that a taphonomic control group will actually be co-preserved with the focal group.

Unfortunately, avian timetree calibration is sensitive to the maximum age constraint selected—the inferred timing of the avian radiation correlates with the selected maximum age constraint of the entire crown group [35]. Estimates for this maximum age constraint have varied from 86.5 Ma [12,16] to 99.6 Ma [11], or even older [14]. Given the topology and DNA branch lengths of the avian tree, fixing the maximum age constraint at 86.5 Ma leads directly to a post-K/Pg avian radiation. This date is based on the age of the Niobrara Formation [37], which has a rich record in terms of the number of fossils, but not in the number of bird taxa [38]. The primary difficulty with this choice is that it is an open marine deposit, which preserves only taxa that fed over the open ocean. As a result, the taxa found there are not suitable taphonomic controls for the vast majority of birds.

In contrast, fixing the maximum age at 94.3 Ma leads to the inference of many Cretaceous-aged modern bird lineages in a recent study [14], but this constraint is also somewhat arbitrary, as it is based on the oldest fossil of the oldest species (*Ichthyornis dispar*) the sister clade to crown-group birds. The first avian NGS study arbitrarily selected an even older maximum age constraint of 99.6 Ma, which nonetheless resulted in a post-K/Pg radiation [11]. A sensitivity analysis indicated that this conclusion would have been different—producing many pre-K/Pg divergences—if the taxon sampling had been increased from approximately 50 to 100 species [14]. We note that in all these analyses, the minimum constraints within crown-group birds are virtually all Cenozoic in age, which further increases the likelihood of finding post-K/Pg divergence times of the living orders.

The challenge with using a maximum age constraint based on the oldest fossils of the sister group is that the origin of the focal group may predate the oldest fossil of the sister group [39]. In the case of Kimball *et al.* [40], 20 out of 24 calibrations have maximum age constraints younger than the K/Pg boundary, with only 2 being slightly older (at 72.6 Ma), essentially guaranteeing the result of a post-K/Pg avian radiation. Kimball *et al.* hard-wired their analysis to yield a post-K/Pg radiation, which cast doubt on the conclusion of Berv *et al.* [41]—based on Kimball *et al.*’s timetree—that the K/Pg event triggered changes in genomic, physiological and life history strategies in birds.

The second approach to account for the incompleteness of the fossil record is gap analysis, which estimate the interval between the oldest known fossil of a lineage (the minimum age estimate discussed above) and its true time of origin, based on the magnitude of temporal gaps between formations that have preserved fossils of the lineage. There are two ways to approach this challenge. The first applies stratigraphic gap confidence intervals to estimate a plausible range for the lineage's time of origin [42–44]. The second, the fossilized birth–death (FBD) process [45], uses the fossil record to infer preservation rates, which are then applied to estimate likely times of origin. When the preservation rate is stochastically constant, both methods yield similar results [36,46]. However, with these approaches we find a range of possibilities—the confidence interval method suggests that the Neoavian radiation occurred largely after the K/Pg mass extinction [13], whereas a more recent analysis using the FBD process suggests that all the deeper nodes of crown-group birds emerged in the Cretaceous, with the Neoavian radiation most likely beginning before the K/Pg boundary [15]. The greatest challenge surrounding gap analysis is that it will underestimate the true times of origin if the preservation potential of the lineage of interest substantially improved over time. This may well have been the case if crown-group bird clades were present but rarer in the Late Cretaceous. Unfortunately, given that nearly all crown-group fossils are Cenozoic in age, the approach has to rely on the gaps of the Cenozoic bird fossil record as a proxy for what might be a sparser Cretaceous crown-group bird fossil record. If the Cenozoic and Mesozoic clade-level fossilization potentials of living clades were similar, then the younger estimates of the avian radiation are probably accurate [13,15], but if the Mesozoic fossilization was substantially less productive (because there were much fewer crown-group species and individuals, for example), then older estimates of the avian radiations become more likely. In this case, Strauss and Sadler's [43] confidence intervals need to be replaced with generalized confidence intervals [47]. For example, when applied to the fossil record of the Caprimulgiformes and assuming a Mesozoic preservation rate one-tenth that of the Cenozoic, the 95% confidence intervals of the time of origin of the group are extended from 68 Ma to approximately 93 Ma [48].

Regardless of the method used, understanding the timing of the radiation of birds depends critically on the fossil record. However, the fossil record can only be employed in a reliable manner when minimum age estimates have been determined with a rigorous approach and when the affinity of the fossils used is not in question.

## NEW BIRD FOSSILS PUSH THE AVIAN DIVERSIFICATION TIMELINE FURTHER INTO THE PAST

This year has seen the discovery of significant early fossils, which have shed new light on the timing of modern bird diversification. Torres *et al.* [26] unearthed a well-preserved Late Cretaceous skull specimen of *Vegavis* from Antarctica that provides a much better understanding of the species’ morphology. Numerous skeletal features now strongly corroborate its placement within Anseriformes, and probably closer to Anatidae than to Anhimidae [26]. In the northern hemisphere, Chen *et al.* [49] reported two new Jurassic bird fossils from Fujian, China. One of these fossils, *Baminornis zhenghensis*, possesses a pygostyle, an advanced feature previously found only in Early Cretaceous birds. The other fossil is a U-shaped furcula from an unknown species, a trait associated with Ornithuromorpha, a lineage that includes crown-group Aves and their close relatives. Both fossils are dated to the Late Jurassic (∼148 Ma), rendering them nearly contemporaneous with the oldest and much more primitive bird *Archaeopteryx* [17,18]. The new Late Jurassic fossils considerably extend the horizon of avian diversification backwards in time. *Baminornis zhenghensis* is now the earliest known bird exhibiting a pygostyle, pushing back the fossil record of pygostyles by approximately 18 million years (a range increase of ∼14%) to the Late Jurassic. In addition, the discovery of the isolated U-shaped furcula from the Late Jurassic, an advanced avian feature that was previously believed to have originated with *Archaeornithura meemannae*, the earliest known Ornithuromorpha from the Early Cretaceous also at ∼130 Ma [20], extends the known stratigraphic range of that feature also by 14%. Although outside of the avian crown group, the Ornithuromorpha are widely assumed to be the radiation that immediately preceded modern Aves [20,21] (Fig. 1).

While formal analyses have yet to be performed, these fossil discoveries suggest that the origin of crown-group Aves may extend at least ∼14% deeper in time than previously assumed (Fig. 1), which may also push the radiation of bird subclades further into the Cretaceous. The geographic dimension of these new discoveries is critical.

**Figure 2.**
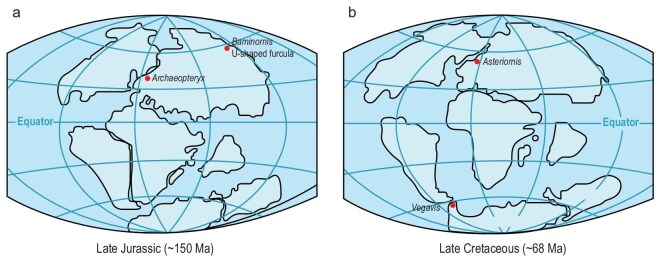
Paleogeographic maps with localities of significant bird fossils. (a) A Late Jurassic map showing the localities of *Archaeopteryx* in Solnhofen (Germany), as well as the newly described *Baminornis zhenghensis* with the first pygostyle and the unknown species with the first U-shape furcula in Fujian (China). (b) A Late Cretaceous map showing the localities of *Vegavis* in Antarctica and *Asteriornis* in Belgium, Europe.

The geographic dimension of these new discoveries is also informative. The discovery of *Baminornis*, an early bird equipped with a pygostyle, along with an unknown species featuring a U-shaped furcula in southern China—at a time when *Archaeopteryx* was extant in Europe—points to a worldwide distribution of stem-group birds in the Late Jurassic, challenging the argument that their emergence was in its earliest stages at the time (Fig. 2a). Similarly, the confirmation of two crown-group Aves from the Late Cretaceous in both the northern and southern hemispheres underscores the widespread distribution of modern birds during that period (Fig. 2b). Collectively, these fossil discoveries suggest a deeper origin of birds and their crown groups than previously hypothesized.

A Mesozoic origin of crown-group birds and their initial radiation in the Late Cretaceous would align their evolutionary trajectories with those of many other major groups [14,27–31,33,34]. The K/Pg boundary catastrophe caused widespread extinction, leading to the demise of entire organismal groups, but many lineages that were already diversifying weathered the storm [33]. When interpreted in this light, multiple macroevolutionary factors beyond the end-Cretaceous catastrophe would have played a significant role in the early diversification of birds [14].
